# Identifying the Physical Fitness and Health Evaluations for Police Officers: Brief Systematic Review with an Emphasis on the Portuguese Research

**DOI:** 10.3390/biology11071061

**Published:** 2022-07-15

**Authors:** Luís M. Massuça, Vanessa Santos, Luís F. Monteiro

**Affiliations:** 1 ICPOL Research Centre, Higher Institute of Police Sciences and Internal Security, 1300-352 Lisbon, Portugal; vstsantos@gmail.com (V.S.); luismonteiro1955@gmail.com (L.F.M.); 2 CIDEFES, Lusófona University, 1749-024 Lisbon, Portugal; 3 CIPER, Faculty of Human Kinetics, University of Lisbon, 1495-751 Cruz Quebrada, Portugal

**Keywords:** tactical, law enforcement, cardiorespiratory fitness, muscular strength, muscular endurance

## Abstract

**Simple Summary:**

Police health and physical fitness are essential for improving quality of life and police skills. This review aims to identify and analyze international and Portuguese studies that have investigated the relationships between various aspects of physical fitness as specified by job descriptions and to understand the health-related requirements of police officers. This will help to select the most used fitness measures and health-related parameters for police officers and improve training curricula for these occupational groups.

**Abstract:**

This review aims (i) to identify and analyze the most used physical fitness tests for police officers (from international and Portuguese studies) and (ii) to understand the health-related physical fitness requirements according to the job descriptions of police officers. A total of 29 studies were included. Eighteen were from around the world and eleven were related to Portuguese police officers. All studies showed acceptable methodological quality in the assessment of physical fitness, and the most used fitness components were muscular strength, endurance, power, aerobic and anaerobic capacity, flexibility, and agility. For the analysis of health parameters, they are insufficient at the international level, while at the Portuguese level we have an acceptable sample. We try to analyze the relationship between physical fitness and health, but the studies conducted so far are insufficient. This review provides summary information (i) to help select the most used fitness measures and health-related parameters for police officers, and (ii) that will serve as a starting point for evaluating the relationship between the health and physical fitness of police officers.

## 1. Introduction

Law enforcement can be a physically demanding, dangerous, and stressful profession that has health implications. The health and physical fitness of police officers is essential to their performing their duties well [1,2].

Police officers must undergo various physical tasks that include carrying external loads, such as running, restraining offenders, self-defense, and manual handling tasks [3]. They also have social and psychological obligations (e.g., the daily pace of work, job responsibilities, and stress/risk situations). It appears that the physically demanding jobs require high levels of cardiovascular fitness as well as muscular strength and endurance [3,4,5].

The field of public safety has high physical fitness and health requirements for entry into police academies. While recruits learn the physical challenges of the profession while being taught the necessary procedures, skills, and the values and behaviors expected of a police officer, these health-related attributes are only assessed for entry and rarely thereafter [6].

According to the literature, a police officer’s physical fitness tends to decline over time. Previous studies suggest that improving traditional health-related components of physical fitness (i.e., body composition, cardiorespiratory fitness, muscular strength, endurance, and flexibility) is essential for improving quality of life and policing skills [7,8]. However, the risk of developing health problems increases with overall decline in physical activity and associated decline in physical fitness. In fact, low levels of muscle fitness and physical endurance as well as overweight and obesity have been shown to be risk factors for police officer health and to lead to lower productivity levels and sick leave [9], resulting in additional costs to the employer. A high prevalence of cardiovascular risk factors has been found among police officers, including metabolic syndrome, hypertension, hyperlipidemia, smoking, and physical inactivity [2].

Studies on the physical activity, physical fitness, and health of Portuguese police officers are scarce. Therefore, this review aims (i) to identify and analyze the most used physical fitness tests for police officers (from international and Portuguese studies) and (ii) to understand the health-related physical fitness requirements according to the job descriptions of police officers.

## 2. Materials and Methods

### 2.1. Experimental Approach to the Problem

A systematic review was conducted to identify the physical fitness tests used on police officers and to describe the fitness levels of this population. This systematic review followed the guidelines of the Preferred Reporting Items for Systematic Reviews and Meta-Analyses (PRISMA) model [10]. This study is exempt from ethical approval because the authors collected and synthesized data from previous studies in which informed consent had already been obtained by the study investigators. Therefore, this study was not approved by an institutional review board.

### 2.2. Procedures

#### 2.2.1. Search Strategy

To identify and obtain relevant original research for the literature review, key literature databases were systematically searched using specific keywords relevant to the topic. The databases searched included PubMed (https://pubmed.ncbi.nlm.nih.gov/?term=police+officer+AND+Physical+Fitness+AND+Health&sort=date, accessed on 28 September 2021), ScienceDirect (https://www.sciencedirect.com/search?qs=Police%20AND%20Fitness%20test%20AND%20health, accessed on 28 September 2021), and ISCPSI (Higher Institute of Police Sciences and Internal Security) common repositories (https://comum.rcaap.pt/handle/10400.26/6300, accessed on 28 September 2021). These databases were selected because they contain a large number of high-quality, peer-reviewed articles and represent journals relevant to the topic of the study. The final search terms and applied filters for the databases searched are summarized in Table 1.

**Figure 1:**
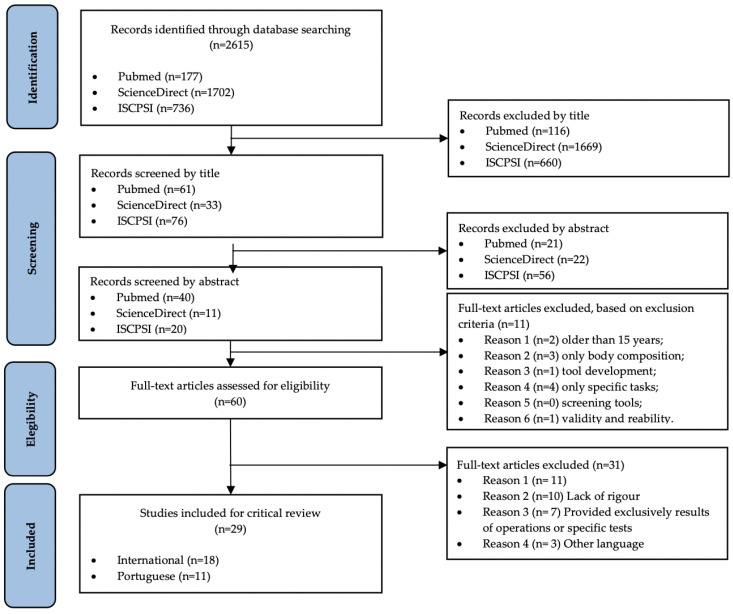
PRISMA diagram detailing the search process.

To improve the relevance of search results, filters that reflected study eligibility criteria were applied in each database when available. In the ISCPSI database, where these filters were not available or were only partially available, eligibility criteria for studies were applied manually by screening study titles and abstracts. The eligibility criteria were then applied to the full text of identified articles that were not excluded during the screening of titles and abstracts to make a final selection of eligible articles for this review. The results of the search, screening, and selection processes were documented in a PRISMA flow diagram (Figure 1) [10]. The inclusion criteria were defined to include individuals from law enforcement, to measure physical fitness, and to measure health. The exclusion criteria were: studies older than 15 years, studies examining only body composition, studies addressing instrument development, studies addressing only weight bearing, studies addressing only screening instruments, validity studies, and reliability studies. Duplicates were removed after all studies were collected.

#### 2.2.2. Critical Appraisal

To assess the methodological quality of the study, the CASP consists of a checklist of ten questions. Each question can be answered “yes,” “can not say,” or “no.” Questions six and seven are short answers that we left blank due to their subjectivity (Critical Appraisal Skills & Programmes, 2018). Methodological quality was also assessed individually by two authors to avoid bias.

#### 2.2.3. Data Extraction

After critically analyzing all articles, we extracted the following information: authors and year of publication; study population; measures (physical fitness tests); measures (health parameters or questionnaires); main results; general conclusions. All of the information is presented in Table 2, Table 3 and Table 4 [2,7,11,12,13,14,15,16,17,18,19,20,21,22,23,24,25,26,27,28,29,30,31,32,33,34,35,36,37]. 

## 3. Results

### 3.1. Search Results

A total of 2615 studies were found during the initial search of the three databases. After removing duplicates and screening by title and abstract, the full-text versions of 60 studies were compiled for review. These studies were then assessed against the inclusion and exclusion criteria, leaving 29 studies for critical review (Table 2 and Table 3). A summary of the screening and selection process, as well as the results of the literature search, can be found in the PRISMA flow diagram [10] (Figure 1). The studies reviewed were related to police candidates, recruits/cadets, or officers. Of the 29 studies, eleven referred to Portuguese police officers [11,12,13,14,15,16,17,18,19,20,21] and the other eighteen referred to police officers from around the world (Brazil, USA, Germany, Canada, Korea, Serbia, and Ireland) [2,7,22,23,24,25,26,27,28,29,30,31,32,33,34,35,36,37]. Eighteen studies examined both male and female participants [7,11,13,15,16,19,21,23,25,26,27,28,30,31,33,34,35,37], while eight studies included only male participants [2,17,18,20,22,24,29,32]. Three studies did not report the gender of the participants [12,14,36].

### 3.2. Fitness Measures

The most commonly used fitness components were muscle strength, endurance and power, aerobic and anaerobic capacity, and some tests of agility and flexibility.

Muscular strength was assessed in nine international articles [7,22,23,24,26,28,29,33,35] and seven Portuguese studies [12,13,15,18,19,20,21], muscular endurance was measured in 16 international articles [7,22,23,24,25,26,27,28,30,31,32,33,34,35,36,37] and seven Portuguese studies [11,12,15,17,19,20,21], and muscular power was measured in 12 international articles [7,22,23,24,26,28,30,31,32,34,35,37] and five Portuguese studies [12,13,19,20,21].

Other measurements of fitness included aerobic capacity, which was assessed in 16 international articles [2,7,22,23,24,25,26,27,28,29,30,31,34,35,36,37] and seven Portuguese studies [11,12,13,17,19,20,21], and anaerobic capacity was assessed in two international articles [23,33] and two Portuguese studies [19,21]. The least commonly reported fitness measures were agility, which was assessed in three international articles [22,23,32] and two Portuguese studies [20,21], and flexibility was assessed in six international articles [22,23,25,27,28,30] with only one test, and four Portuguese studies [12,15,20,21] with two tests.

Maximal muscular strength was measured in all studies in different forms, including one repetition maximum (1RM) bench press [12,19,22,23,24,28] and leg press [22], handgrip strength [7,12,13,15,18,19,20,21,22,23,26,33,35], back-leg–chest strength [21,26], finger-grip strength [18], and elbow flexion [29]. Muscular endurance was most commonly measured by push-ups [11,12,17,19,21,22,23,24,25,26,27,28,30,31,33,34,35,36,37], sit-ups [11,15,17,19,20,21,23,24,25,26,27,28,29,30,31,33,34,35,37], pull-ups [7,11,12,17,20,21,32,34,36], curl-ups [22], plank time [7], and flexed arm hang [26,29]. Muscular power was measured by vertical jump [7,12,13,19,20,22,23,24,26,28,30,31,32], standing long jump [19,20,21,32,35], medicine ball throw [12,20], and mountain climbers [34].

A wide range of aerobic capacity measures was performed, including treadmill-based aerobic testing [2,13,19,22] or estimated *V*O_2max_ (201 m run [34], 300 m run [24,28], 1.5-mile run [24,28,31,34], 2.4 km run [27,30,37], 20 m shuttle run [20,21,25,26,29,31,36], 12 min Cooper [35,36], and arm ergometer assessment [23,37]). Anaerobic capacity was measured using either the Wingate anaerobic test [23], or sprint tests [19,21,23,33,37].

Agility was tested by a change of direction test [20,21,22,23,32], and flexibility was measured by the sit-and-reach test [12,15,20,21,22,23,25,27,29,30] and shoulder flexibility [20].

### 3.3. Health Parameters

Few studies included health parameter assessments or questionnaires in their study design. Anthropometric measurements (classical anthropometry) were the most common, used in 17 international studies [2,7,22,23,24,25,26,27,28,29,30,31,32,33,34,35,37] and in 9 Portuguese studies [11,12,13,14,16,18,19,20,21]. The second most commonly used assessment was the international physical activity questionnaire (IPAQ) [13,14,16,18,19,20,21,22,23,24,25,26,27,28,29,30], i.e., the short form, which was assessed in one international [29] and five Portuguese [14,16,18,19,20] studies. Other assessments used in a few studies were the Framingham risk score [2,13], used in two studies; the Jackson’s questionnaire [15,18,19], used in three studies; and the physical activity readiness questionnaire [19,20], used in two studies. Blood pressure [2,13,25], heart rate [7,19,20,25], blood serum [2,13] and lactate [19,20], injuries [7], fatigue [19], stress [14], quality of life [13], or other factors [2,14,15,16,35] were measured as health parameters in some studies.

The results of this study have important implications for the selection of the most commonly used fitness measures for police officers and for the improvement of training plans for police officers, which we have summarized in a synoptic table (Table 5).

## 4. Discussion

All studies showed acceptable methodological quality in the assessment of physical fitness. The analysis of health parameters is insufficient at the international level, while at the Portuguese level we have an acceptable sample for police health. However, if we try to analyze the relationship between physical fitness and health, the studies conducted so far are insufficient, though we will return to this point later.

Differences in fitness tests and test selection procedures have been noted, highlighting the need for standardization of fitness test procedures to ensure consistency and precision when comparing results [31]. Internationally, the most applied assessments were: (i) push-ups [7,22,23,24,25,26,27,28,30,31,33,34,35,36,37], sit-ups [23,24,25,26,27,28,29,30,31,33,34,35,37], vertical jump [7,22,23,24,26,28,30,31,32], and handgrip test [7,22,23,26,33,35] for muscle strength; (ii) 12 min Cooper [35,36], 1.5-mile run [24,28,31,34], 2.4-mile run [27,30,37], and 20 m shuttle run [25,26,29,31,36] for aerobic capacity; and (iii) sit-and-reach [22,23,25,27,29,30] for flexibility. In Portugal, the most commonly applied assessments were: (i) push-ups [11,12,17,19,20,21] and sit-ups [11,15,17,19,20,21] for muscle strength; (ii) standing long jump [19,20,21], vertical jump [12,13,19,20], and medicine ball throw [12,20] for muscle power; (iii) 12 min Cooper [11,12,17,21] for aerobic capacity; and (iv) sit-and-reach for flexibility [12,15,20,21]. Portuguese police officers showed higher levels of physical activity than the general population [12,14], and, in comparing their data with those of international police officers, they showed intermediate levels [15]. Male police officers performed significantly better than female officers on all measures [20,25,26,30,35].

We believe it is important to standardize the scores for the different physical abilities, using our synoptic table to achieve consistency in the assessment parameters for police function.

Regarding health status in terms of physical fitness, a general decline in certain physical attributes between genders has been observed with age [26]. Aerobic capacity emphasizes the need for physical training in order not to compromise performance and to mitigate the effects of increasing age [17,22]. Several studies have shown that the increase in body fat percentage is associated with a decrease in performance and physical fitness [24,27,28]. An increase in lean body mass and a decrease in body fat percentage can positively affect vertical jump performance [24]. Higher body fat percentage resulted in lower cardiorespiratory capacity, lower dynamic strength, and lower flexibility [27]. Moreover, it was proved that improving metabolic fitness and muscular endurance should be the goal of conditioning to improve sit-up performance and running times [23,24]. Agility, aerobic capacity, push-ups, and sit-ups were significantly correlated with police officer tasks [22].

The development of health-related fitness standards and associated health and fitness strategies will help improve officer health and fitness. A strong correlation was found between the morphological, cardiorespiratory, and neuromuscular components of health-related physical fitness [29]. Physical fitness (including anthropometric measures) and health measures should be used together to guide conditioning interventions to improve police performance [12,13,19,22,24,35].

Analysis of these studies with police officers confirms that physical fitness is extremely important for the performance of operational tasks and has a direct influence on health status. Body composition showed a direct influence on physical fitness and cardiovascular risk. In addition, decreased cardiorespiratory fitness was associated with an increase in age-related cardiovascular and metabolic risk [13,14,15,21,22,28,30,34,37]. However, there is a need to implement health-promotion interventions to address cardiovascular and metabolic risk factors [37]. Several studies have found a significant association between age and decline in physical fitness [12,13,15,16,17,21,22,26]. It is critical that police officers maintain appropriate levels of physical fitness as they age [22,26].

However, a limitation of this review was that it was not possible to screen studies that reported on physical fitness and its relationship with health parameters. Few clinical parameters were evaluated to critically analyze this risk relationship. Furthermore, the great variety of physical fitness assessments studied can also be considered a limitation, as the diversity of tests makes it difficult to standardize the protocol for fitness assessment. Another limitation was the inclusion of studies with cadets/recruits and cadets who are not yet police officers.

## 5. Conclusions

The police profession involves special challenges to the health, physical, and psychological statuses of police officers. The risks of performing police work have numerous, complex, and long-lasting consequences that affect not only the quality of everyday life of police officers, but also the efficiency of the measures and activities undertaken. Therefore, it is necessary to maintain physical condition at an optimal level over a long period of time, monitor changes in the health status of police officers, and point out in a timely manner the positive and negative implications of irresponsible attitudes towards these issues by police officers and police management.

In fact, our research shows that a variety of physical fitness tests exist to assess and predict police officer performance. More and more tests are being used to assess various physical abilities, such as muscular strength and aerobic capacity, but agility and flexibility are still poorly assessed. However, health-related tests are rarely used as a complementary method to diagnose physical and health conditions, even though it is known that there is a direct relationship between the two.

For such a research endeavor, the existing work is a good starting point; the literature referred to also indicates possible directions for research engagement (e.g., researching correlations between regular physical activity, efficiency of police work, and monitoring changes in police health status), and the contextual framework provides an opportunity to identify and use key determinants that shape the health quality of police officers. Accordingly, efforts should be made to evaluate the same protocol of physical fitness tests and include health parameters and to use the results obtained to improve training plans for this occupational group.

## Figures and Tables

**Table 1:** Databases and relevant search terms.

Databases	Search Terms	Filters (Sort By)	Results
PubMed	“Police” OR “Law enforcement” AND “Fitness test” OR “Physical fitness” AND “heath”	Best Match	177
ScienceDirect	“Police” AND “Fitness test” AND “heath”	Relevance	1702
ISCPSI ^1^	Dissertations Scientific Activity Final Research Papers—Police Command and Direction Course Final Research Papers—Police Direction and Strategy Course	All	736

^1^ Higher Institute of Police Sciences and Internal Security (Lisbon, Portugal) common repository.

**Table 2:** Data extraction table, including fitness and health measures, with key findings-International Research.

Author/Year of Publication	Population	Measures Physical Fitness Tests	Measures Health Parameters or Questionnaires	Main Results/General Conclusions
Beck et al., 2015 [22]	*n* = 16 (male) USA Law Enforcement Officers	▪ 1 RM bench press ▪ 1 RM leg press ▪ Handgrip ▪ Vertical jump ▪ Push-ups ▪ Curl-ups ▪ Agility ▪ Sit-and-reach ▪ *V*O_2max_ (treadmill) ▪ Police ability test	▪ CS anthropometry ▪ DXA	▪ Age correlated significantly with most police ability tests, physical fitness, and anthropometric assessments. ▪ Push-ups, curl-ups, body mass, waist circumference, and abdominal circumference correlated significantly with individual police ability tests. ▪ Training programs should focus on managing body composition and fitness in older police officers.
Crawley et al., 2016 [23]	*n* = 55 (male, *n* = 49; female, *n* = 6) Michigan, USA Police Cadets	▪ Speed (40-yard) ▪ Handgrip ▪ 1 RM bench press ▪ Push-ups ▪ Sit-ups ▪ Vertical jump ▪ Half-mile shuttle run ▪ Arm ergometer ▪ Agility (*t*-test) ▪ Sit-and-reach ▪ Wingate (30 s)	▪ CS anthropometry ▪ SK measurements	▪ Significant changes were noted in agility, peak upper and lower body power, sit-ups, and push-ups during the first 8 weeks, and in agility, peak lower body power, sit-ups, push-ups, and half-mile commute throughout the 16 weeks. ▪ The cadets were able to pass the required state fitness tests at the end of the academy; the training programs could easily benefit from proper periodization.
Dawes et al., 2016 [24]	*n* = 76 (male) Police Officers	▪ 1 RM bench press ▪ Sit-ups ▪ Push-ups ▪ Vertical jump ▪ 300 m run ▪ 1.5-mile run ▪ *V*O_2max_ (predicted)	▪ CS anthropometry ▪ SK measurements	▪ An increase in body fat percentage is associated with a decrease in performance. ▪ Estimated lean body mass was significantly and positively correlated with push-ups, 1RM bench press, and vertical jump, whereas an increase in estimated fat mass was significantly associated with decreased performance in sit-ups, vertical jump, 1.5-mile run, and estimated *V*O_2max_. ▪ Reducing body fat mass while improving metabolic fitness and muscular endurance should be the goal of conditioning training to improve performance in sit-ups and running times (over short or longer distances). ▪ Increasing lean body mass and decreasing body fat mass can both positively affect vertical jump performance.
Losty et al., 2016 [25]	*n* = 273 (male, *n* = 188; female, *n* = 85) Irish Police Officers Trainees	▪ Push-ups ▪ Sit-ups ▪ 20 m shuttle run ▪ *V*O_2max_ (predicted) ▪ Sit-and-reach	▪ CS anthropometry ▪ BIA ▪ Blood pressure ▪ Heart rate	▪ The mean value of sit-ups of 22 was considered poor (for men and female). ▪ The estimated *V*O_2max_ of 42 was considered mediocre for a male and good for a female compared to the norms. ▪ The values for push-ups and sit-ups also improved. ▪ The physiological values of the female participants decreased more significantly than those of the male participants. ▪ This study provided foundational information to set meaningful fitness standards for this unique Irish workforce, from recruiting future trainees to setting thresholds and guidelines for future fitness testing.
Dawes et al., 2017 [26]	*n* = 631 (male, *n* = 597; female, *n* = 34) Highway Patrol Officers	▪ Handgrip ▪ Push-ups ▪ Sit-ups ▪ Back-leg–chest strength ▪ Vertical jump ▪ 20 m shuttle run	▪ CS anthropometry	▪ Significant differences between genders in all anthropometric and fitness measures (most consistent in the 30–39 age group). ▪ In female, there was a general (nonsignificant) decrease in push-up and pendulum running performance. ▪ For men, there were significant differences between the 20–29 age group and the 30–39, 40–49, and 50–59 age groups (with the younger group performing better on the vertical jump, push-ups, sit-ups, and pendulum run). ▪ There were no differences between age groups in handgrip and back-leg–chest strength.
Violanti et al., 2017 [27]	*n* = 1941 (male, *n* = 1826; female, *n* = 115) USA Police Officers	▪ Push-ups ▪ Sit-ups ▪ 2.4 km run ▪ Sit-and-reach	▪ SK measurements	▪ Percent body fat mass was linearly and positively related to time of 2.4 km run and linearly and inversely related to number of push-ups, sit-ups, and sit-and-reach in males, and similar associations were observed in females, except for sit-and-reach. ▪ Percent body fat mass was inversely associated with fitness level in men and female. ▪ Officers with a higher percentage of body fat mass had lower cardiorespiratory capacity, lower dynamic strength, and lower flexibility.
Orr et al., 2018 [28]	*n* = 164 (male, *n* = 139; female, *n* = 25) *n* = 84 (male, *n* = 66; female, *n* = 18) Police Academy Cadets *n* = 80 (male, *n* = 73; female, *n* = 7) Police Officers	▪ Push-ups ▪ Sit-ups ▪ 1 RM bench press ▪ Vertical jump ▪ 300 m run ▪ 1.5-mile run	▪ CSl anthropometry ▪ SK measurements	▪ Male cadets had significantly lower body fat mass than male officers, which can be explained by age differences between these groups. ▪ Male cadets were more aerobically fit and had greater muscular endurance than male officers, regardless of age. ▪ Male cadets also had greater anaerobic fitness than male officers, although age differences only partially explained this difference. ▪ Female cadets exhibited higher upper body strength and muscular endurance than female officers, regardless of age. ▪ Individualized training approaches that target the observed increase in body fat mass associated with aging would be beneficial for police officers, whether they are cadets or on-duty officers.
Lima-dos-Santos et al., 2018 [29]	*n* = 47 (male) Brazilian *n* = 25 Special Police Operations *n* = 22 Traffic Police	▪ Sit–ups ▪ Elbow flexion test ▪ 20 m shuttle run ▪ Sit-and-reach ▪ *V*O_2max_ (predicted)	▪ CS anthropometry ▪ SK measurements ▪ IPAQ-S	▪ Both groups showed moderate to strong correlations between waste circumference, BMI, and body fat mass percentage and local muscle resistance and dynamic muscle strength. ▪ Significant correlations were found between *V*O_2max_, waste circumference, BMI, body fat mass percentage, and local muscle resistance, but not with flexibility. ▪ Strong correlation between health-related physical fitness and morphological, cardiorespiratory, and neuromuscular components, except flexibility, in a separate analysis of special police units.
Lockie et al., 2019 [30]	*n* = 383 (male, *n* = 362; female, *n* = 21) USA Law Enforcement Officers	▪ Push-ups ▪ Sit-ups ▪ Vertical jump ▪ 2.4 km run ▪ Sit-and-reach	▪ CS anthropometry	▪ Sit-and-reach performance of this sample compared well with similar populations, and adequate levels were maintained with increasing age. The 60 s push-up performance did not vary with age. ▪ Older females should attempt to maintain or improve their relative upper body strength and strength endurance. ▪ Age-related deterioration of cardiorespiratory function. ▪ Upper body and abdominal strength should be trained in appropriate resistance training programs. ▪ Improving maximal strength and endurance capabilities of the abdominal muscles and core region is important to reduce the risk of lower back pain. ▪ Lower body strength and sprint training should be performed at all ages to improve or maintain leg muscle performance. ▪ Strength and conditioning coaches must ensure that aerobic capacity is sufficient to successfully meet the specific demands of their profession.
Myers et al., 2019 [31]	*n* = 398 (male and female) USA G1, *n* = 79 G2, *n* = 319 Law Enforcement Agencies	▪ Push-ups ▪ Sit-ups ▪ Vertical jump ▪ 1.5-mile run ▪ 20 m shuttle run ▪ *V*O_2max_ (predicted)	▪ CS anthropometry	▪ G1 performed significantly better than G2 in push-ups, sit-ups, vertical jump, and *V*O_2max_. ▪ In men, G1 performed significantly better than G2 in push-ups, sit-ups, and vertical jump performance. ▪ Fitness standards and exercise protocols need to be developed and tailored to the specific population and needs of each law enforcement agency. ▪ Differences in fitness testing procedures were noted, highlighting the need for standardization of fitness testing procedures to ensure consistency and accuracy when comparing results.
Frio Marins et al., 2019 [32]	*n* = 13 (male) Brazilian Federal Highway Police Officers	▪ Vertical jump (SJ, CMJ) ▪ Standing long jump ▪ Pull-ups ▪ Illinois agility test ▪ *V*O_2max_ ▪ Biering Sorensen Test ▪ Police ability test	▪ CS anthropometry	▪ Agility, lower limb power, and height were significantly related to performance in police ability tests without load carrying. ▪ Aerobic power, lower limb power, and agility were significantly related to performance in the police ability test with load carrying. ▪ The best predictors of performance in the police ability test were agility without load carrying and *V*O_2max_, upper limb strength, and agility with load carrying. ▪ Therefore, training programs to improve police occupational performance considering load carrying should aim to improve agility, upper limb strength, and aerobic fitness.
Lentz et al., 2019 [7]	*n* = 1006 (male and female) Canada Police Officers	▪ Handgrip ▪ Pull-ups ▪ Push-ups ▪ Plank time ▪ Vertical jump ▪ *V*O_2max_	▪ CS anthropometry ▪ Injuries ▪ Heart rate	▪ Significant differences in fitness test scores between injured and uninjured subjects on all measures except: body fat mass percentage, body mass, HR, left handgrip, and combined handgrip strength. ▪ Age, gender, vertical jump height, leg power, number of pull-ups and push-ups, kilograms pulled, and *V*O_2max_ were significantly related to musculoskeletal injury incidence. ▪ The results suggest an interaction between gender and *V*O_2max_, such that the effect of *V*O_2max_ on injury risk cannot be understood without accounting for gender. ▪ The relationship between occupation-specific fitness test performance and work-related injuries will provide new insights for prevention strategies.
Kim and Kim, 2019 [33]	*n* = 372 (male, *n* = 334; Female, *n* = 38) Korean Police Officers	▪ Speed (100 m) ▪ Handgrip ▪ Push-ups ▪ Sit-ups	▪ CSl anthropometry ▪ BIA	▪ Performance in the 100 m sprint, push-ups, and sit-ups were lower in 2019 compared to previous years. ▪ The 100 m sprint times (only) of female showed a statistically significant difference between years. ▪ The physical fitness of Korean police officers decreased during the year. ▪ In particular, the physical fitness of men decreased.
Lockie et al., 2020 [34]	*n* = 908 (male, *n* = 761; female, *n* = 147) USA Law Enforcement Officers	▪ Push-ups ▪ Sit-ups ▪ Mountain climbers ▪ Pull-ups ▪ 201 m run ▪ 1.5-mile shuttle run test	▪ CS anthropometry	▪ No significant differences in age or body mass between classes. ▪ Physical fitness of police recruits may vary among classes in the academy, i.e., performance in push-ups, mountain climbers, 201 m run, and 2.4 km run differed among classes. ▪ Normative percentile ranking data showed large differences between recruits. ▪ Females were at the bottom of the percentile ranges for all assessments. ▪ It is recommended that law enforcement training personnel use fitness assessment data to guide their physical training program. ▪ Fitness assessment data can be used to identify strengths and weaknesses of recruits so that specific fitness qualities that may be helpful for future job performance can be improved.
Kukić et al., 2020 [35]	*n* = 177 (male, *n* = 98; female, *n* = 79) Serbia Police Students	▪ Handgrip ▪ Standing long jump ▪ Push-ups ▪ Sit-ups (30 s) ▪ Cooper	▪ CS anthropometry ▪ PSDQ-S	▪ Perceived strength and endurance correlated with handgrip, standing long jump, sit-ups, 12 min Cooper run, and BMI. ▪ When analysed by gender, (i) perceived strength correlated only with handgrip and BMI in males and sit-ups in females, whereas (ii) perceived endurance correlated with 12 min Cooper performance in both genders. ▪ Improved precision of physical self-concept could increase awareness of physical self and objectivity of perception of physical performance. This could be relevant to exercise behaviour, as police students could use more precise strength and conditioning programmes in their free time aimed at demonstrating specific components of fitness.
Caetano et al., 2021 [36]	*n* = 1705 Paraná, Brazil Military Police	▪ Pull-ups ▪ Push-ups ▪ Flexed-arm hang ▪ Shuttle run ▪ Cooper	-	▪ When comparing 2016, 2017, 2018, and 2019, significant differences were found for all physical fitness variables. ▪ The mean scores for the shuttle run and upper body test were highest in 2019, while the mean score for the 12 min run test was highest in 2017 (small effect size). ▪ The addition of the physical fitness test as a requirement for promotion in 2016 was associated with an improvement in the physical fitness of field officers and likely played a causal role in this change.
Lockie et al., 2021 [37]	*n* = 514 (male and female) *n* = 436 Graduate Recruits *n* = 78 Recruit not Complete	▪ Speed (75-yard) ▪ Push-ups ▪ Sit-ups ▪ Arm ergometer ▪ 2.4 km run	▪ CS anthropometry	▪ Push-ups, 75-yard run, arm ergometer, and 2.4 km run generally improved. ▪ Recruits tended to perform worse on the fitness tests compared with university graduates. ▪ Both recruits showed some improvement in fitness from initial recruitment to the academy. ▪ The group that did not take the recruit test had lower muscular endurance, running speed, and aerobic capacity during recruitment and did not improve significantly until entering the academy, which likely affected academy survival.
Strauss et al., 2021 [2]	*n* = 55 (male) German Police Officers	▪ *V*O_2max_ (treadmill)	▪ CS anthropometry ▪ BIA ▪ Blood pressure ▪ Blood serum ▪ Framingham risk ▪ Questionnaire sedentary time at work	▪ Participants had a high prevalence of pre-obesity. ▪ Participants had a high prevalence of abnormal triglyceride levels and systolic and diastolic blood pressure levels. ▪ The average 10-year cardiovascular risk (according to Framingham) was considered moderate. ▪ Metabolic syndrome was diagnosed in 32% of participants. ▪ This study showed increased cardiovascular and metabolic risk and lower cardiorespiratory fitness in German police officers. ▪ There is a need for health-promoting measures and concepts, such as company sports or nutrition courses, to counteract cardiovascular and metabolic risk factors.

BIA, bioelectrical impedance analysis; BMI, body mass index; CS anthropometry, classical anthropometry; DXA, dual-energy X-ray absorptiometry; CMJ, countermovement jump; HR, heart rate; IPAQ-S, International Physical Activity Questionnaire—Short Form; PSDQ-S, Physical Self-Description Questionnaire—Short Form; SK measurements, skinfold thickness measurements; SJ, squat jump; *V*O_2max_, aerobic capacity.

**Table 3:** Data extraction table, including fitness and health measures, with key findings-Portuguese Research.

Author/Year of Publication	Population	Measures Physical Fitness Tests	Measures Health Parameters or Questionnaires	Main Results/General Conclusions
Araújo et al., 2021 [12]	*n* = 117 Portuguese Police Officers (Special Police Unit)	▪ Handgrip ▪ 1 RM bench press ▪ Medicine ball throw (3 kg) ▪ Squat Jump ▪ Pull-ups (max.) ▪ Push-ups (2 min) ▪ Sit-and-reach ▪ Cooper	▪ CS anthropometry ▪ BIA	▪ Annual age losses were found in physical fitness, namely, strength: left handgrip strength, bench press, squat jump, medicine ball throw, push-ups, pull-ups, sit-ups, and *V*O_2max_. ▪ Participants exhibited good physical fitness status consistent with the demands of their occupation. ▪ Regardless of the effects of age, they were able to maintain good fitness and very good aerobic performance over the years. ▪ Loss of strength was most strongly associated with age.
Sá et al., 2021 [11]	*n* = 32 (male and female) Portuguese Police Officers	▪ Pull-ups ▪ Sit-ups ▪ Push-ups ▪ Cooper	▪ CS anthropometry ▪ DXA	▪ Mean scores were also lower compared with elite police officers in an older age group in Portugal and compared with regular police officers of similar age from Portugal and from the United States. ▪ Participants reported higher average values for pull-ups, sit-ups, and push-ups than elite police officers in Portugal who were in an older age group. ▪ For *V*O_2max_ prediction, participants reported lower average values than elite Portuguese police officers who were in an older group and within the interval average range identified in a recent systematic review. ▪ The participants’ mean systolic blood pressure values were categorized as “pre-hypertensive” and diastolic blood pressure as “normal”. ▪ The Portuguese recruits were found to be extremely fit and had robust physical fitness and anthropometric profiles, as well as good metabolic indicators. ▪ In addition, the study would contribute to the development and implementation of regular exercise and training programs aimed at maintaining high levels of physical fitness throughout the career and lifespan of police officers, thus optimizing their deployment, with a potential positive impact on overall health.

BIA, bioelectrical impedance analysis; CS anthropometry, classical anthropometry; DXA, dual-energy X-ray absorptiometry; *V*O_2max_, aerobic capacity.

**Table 4:** Data extraction table, including fitness and health measures, with key findings-Portuguese Research (Master’s Thesis).

Author/Year of Publication	Population	Measures Physical Fitness Tests	Measures Health Parameters or Questionnaires	Main Results/General Conclusions
Jerónimo Pina 2012 [13]	*n* = 1038 (male and female) Portuguese Police Officers	▪ Handgrip ▪ Vertical jumps (SJ; CMJ) ▪ Aerobic capacity (YYIR) ▪ *V*O_2max_ (treadmill)	▪ CS anthropometry ▪ Blood pressure ▪ Blood serum ▪ Framingham risk ▪ IPAQ-L ▪ SF-36v2	▪ Significant effects of biosocial characteristics (age, occupation, occupational function) on physical activity, physical activity on quality of life and Framingham risk, quality of life dimensions on metabolic syndrome, and physical activity on physical fitness, especially anaerobic threshold. ▪ Association between anaerobic threshold and two quality of life dimensions, although there was no association between physical fitness and metabolic syndrome and Framingham risk. ▪ Physical activity showed a direct effect on metabolic syndrome and Framingham risk.
Catarina Silva Batista 2014 [14]	*n* = 245 Portuguese Police Officers	-	▪ CS anthropometry ▪ Fantastic lifestyle questionnaire ▪ Stress vulnerability scale ▪ IPAQ-S	▪ IPAQ-S: 79% high; 14% moderate; 7% low. ▪ Physical activity has a positive effect on lifestyle and stress reduction. ▪ A positive relationship between physical activity and lifestyle; on the other hand, there is no relationship between the level of physical activity and susceptibility to stress.
João Prisciliano 2014 [15]	*n* = 406 (male and female) Portuguese Police Officers	▪ Sit-and-reach ▪ Handgrip ▪ Sit-ups	▪ Jackson questionnaire ▪ Physical health questionnaire ▪ Work ability index	▪ PSP elements have higher levels of physical fitness than the general population. Compared to international tables, they have an intermediate level of physical fitness. ▪ Age is a characteristic highly related to the decrease in physical fitness level, especially in the *V*O_2max_ component, abdominal strength, and handgrip in both hands. ▪ Physical health status decreased with age. Work capacity decreases with age. Physical health status is closely related to cardiorespiratory capacity, abdominal strength, and handgrip strength, of both hands and body mass values. ▪ Those who have better physical fitness have better condition and health, and thus better working capacity.
Sérgio Paulo 2015 [16]	*n* = 933 (male, *n* = 861; female, *n* = 72) Portuguese Police Officers	-	▪ CS anthropometry ▪ IPAQ-S ▪ Food frequency questionnaire ▪ Pittsburgh sleep quality index	▪ IPAQ-S: 40.3% high; 48.8% moderate; 10.9% low. ▪ As we age, physical activity and dietary intake decrease, with no significant change in body composition. ▪ Plans should be implemented to increase physical activity and improve dietary habits to improve health and fitness for critical police situations.
Frederico Belchior 2015 [17]	*n* = 1747 (male) Portuguese Police Officers (Special Police Unit)	▪ Pull-ups ▪ Push-ups ▪ Sit-ups ▪ Cooper ▪ Police ability test	-	▪ Performance in physical fitness and policing ability tests decreased significantly with increase in age group. ▪ Aerobic capacity was negatively and significantly related to time spent performing the police ability test. ▪ The effect of aerobic capacity in the police ability test underscores the need for physical training of these police officers (throughout their careers) so as not to impair their performance, while mitigating the effects of increasing age.
Carlos Carvalho 2016 [18]	*n* = 81 (male) Portuguese Police Officers	▪ Handgrip ▪ Finger-grip	▪ CS anthropometry ▪ IPAQ-S ▪ Jackson questionnaire	▪ IPAQ-S: 72.8% high; 25.9% moderate; 1.2% low. ▪ The intervention team had higher levels of physical performance as measured by *V*O_2max_. ▪ With increasing age, physical activity level, physical performance and shooting performance decrease significantly. ▪ Significant and negative relationship between age, physical activity level, physical capacity, and shooting performance, with an obvious dominance of cardiorespiratory capacity. ▪ The model for shooting performance had age, body mass index, physical activity level, and cardiorespiratory capacity as explanatory variables, and this variation was explained 48%. ▪ The effects of cardiorespiratory capacity, body mass index, age, and physical activity level on shooting performance underscore the need for training for emergency officers to prevent degradation of job performance efficiency while minimizing the effects of age.
João Teixeira 2017 [19]	*n* = 97 (male, *n* = 97; female, *n* = 4) Portuguese Police Officers	▪ Handgrip ▪ Vertical jump (CMJ) ▪ Push-ups ▪ Sit-ups ▪ Standing long jump ▪ 1RM Bench Press ▪ RAST ▪ *V*O_2max_ (treadmill) ▪ Police ability test	▪ CS anthropometry ▪ BIA ▪ IPAQ-S ▪ Jackson questionnaire ▪ PAR-Q ▪ Fatigue ▪ Heart rate ▪ Lactate	▪ IPAQ-S: 69.1% high; 25.8% moderate; 5.2% low. ▪ Police officers’ overall fitness test times showed strong and significant correlations with their general physical fitness test results in the field. ▪ Performance can be predicted using general physical fitness tests.
Pedro Oliveira 2021 [20]	*n* = 42 (male) Portuguese Police Officers (Special Police Unit)	▪ Handgrip ▪ Agility (*t*-test) ▪ 20 m shuttle run ▪ Sit-ups ▪ Pull-ups ▪ Medicine ball throw ▪ Standing long jump ▪ Vertical jump (CMJ) ▪ Sit-and-reach ▪ Shoulder flexibility ▪ Police ability test	▪ CS anthropometry ▪ SK measurements ▪ Heart rate ▪ Lactate ▪ IPAQ-S ▪ PAR-Q	▪ IPAQ: 87.9% high; 12.1% moderate; 0% low. ▪ The elite police officers had good fitness levels and body composition, also compared with the international police panorama. ▪ There was also a strong negative correlation between fitness variables and time spent on the circuit, i.e., the higher the fitness level, the shorter the time spent performing the circuit for on-duty tasks. ▪ The pull-ups, agility test performance, and right-handgrip values explained the variation in performance. ▪ The use of personal protective equipment significantly degrades the performance of elite police officers and interferes with their police work.
Gabriel Coutinho 2021 [21]	*n* = 686 (male, *n* = 555; female, *n* = 131) Portuguese Police Cadets	▪ Speed (30 m; 60 m) ▪ Agility (slalom) ▪ Standing long jump ▪ Sit-ups (60 s) ▪ Pull-ups ▪ Push-ups ▪ Back-leg–chest strength ▪ Handgrip ▪ Sit-and-reach ▪ Cooper ▪ 20 m shuttle run	▪ CS anthropometry	▪ Female performance was lower than male performance in physical tests. ▪ Throughout the course, physical condition was maintained/improved, except for aerobic capacity in males. ▪ As age group increased, performance in physical fitness tests tended to decrease for both genders (except for handgrip and back-leg–chest strength). ▪ The police academy course managed to maintain the physical fitness of the cadets. ▪ The 20–29 age group is the one with the best physical fitness for both genders. ▪ Cadets older than 29 show the greatest drop-off in performance in physical fitness tests for both genders.

BIA, bioelectrical impedance analysis; CS anthropometry, classical anthropometry; CMJ, countermovement jump; IPAQ, International Physical Activity Questionnaire (IPAQ-L, Long Form; IPAQ-S, Short Form); PAR-Q, Physical Activity Readiness Questionnaire; RAST, running based anaerobic sprint test; SF-36v2, Health Survey; SK measurements, skinfold thickness measurements; SJ, squat jump; YYIR, Yo-Yo intermittent recovery test; *V*O_2max_, aerobic capacity.

**Table 5:** Synoptic table.

	Fitness Tests	Assessed Physical Capacity	References	Major Conclusions
Most used tests internationally	Push-ups (in 15 articles)	Muscular endurance	[7,22,23,24,25,26,27,28,30,31,33,34,35,36,37]	The most commonly used tests to evaluate: Muscular endurance: push-ups, sit-ups, or pull-ups; Muscular power: vertical jump, standing long jump, or medicine ball throw; Muscular strength: handgrip or 1RM bench press. Aerobic capacity: 1.5-mile or 20 m shuttle run test, or Cooper test. Flexibility: sit-and-reach. Agility: t-test.
	Sit-ups (in 13 articles)	Muscular endurance	[23,24,25,26,27,28,29,30,31,33,34,35,37]	
	Vertical jump (in 9 articles)	Muscular power	[7,22,23,24,26,28,30,31,32]	
	Handgrip (in 6 articles)	Muscular strength	[7,22,23,26,33,35]	
	Sit-and-reach (in 6 articles)	Flexibility	[22,23,25,27,29,30]	
	20 m shuttle run test (in 5 articles)	Aerobic capacity	[25,26,29,31,36]	
	1.5-mile shuttle run test (in 4 articles)	Aerobic capacity	[24,28,31,34]	
	1 RM bench press (in 4 articles)	Muscular strength	[22,23,24,28]	
	Pull-ups (in 4 articles)	Muscular endurance	[7,32,34,36]	
	Agility t-test (in 2 articles)	Agility	[22,23]	
Most used tests in Portugal	Handgrip (in 7 articles)	Muscular strength	[12,13,15,18,19,20,21]	
	Sit-ups (in 6 articles)	Muscular endurance	[11,15,17,19,20,21]	
	Push-ups (in 5 articles)	Muscular endurance	[11,12,17,19,21]	
	Pull-ups (in 5 articles)	Muscular endurance	[11,12,17,20,21]	
	Cooper test (in 4 articles)	Aerobic capacity	[11,12,17,21]	
	Sit-and-reach (in 4 articles)	Flexibility	[12,15,20,21]	
	Vertical jump (in 4 articles)	Muscular power	[12,13,19,20]	
	Standing long jump (in 3 articles)	Muscular power	[19,20,21]	
	Medicine ball throw (in 2 articles)	Muscular power	[12,20]	
	1 RM bench press (in 2 articles)	Muscular strength	[12,19]	
	20 m shuttle run test (in 2 articles)	Aerobic capacity	[20,21]	
	Agility t-test (in 1 article)	Agility	[20]	

## Data Availability

Not applicable.
